# Mixotrophic Cultivation of *Dunaliella tertiolecta* in Cheese Whey Effluents to Enhance Biomass and Exopolysaccharides (EPS) Production: Biochemical and Functional Insights

**DOI:** 10.3390/md23030120

**Published:** 2025-03-11

**Authors:** Konstantina Tsotsouli, Spyros Didos, Konstantinos Koukaras, Anagnostis Argiriou

**Affiliations:** 1 Institute of Applied Biosciences, Centre for Research and Technology Hellas, Thermi, 57001 Thessaloniki, Greece; ktsotsouli@certh.gr (K.T.); sdidos@certh.gr (S.D.); kkoukaras@certh.gr (K.K.); 2 Department of Food Science and Nutrition, University of the Aegean, Myrina, 81400 Lemnos, Greece

**Keywords:** microalgae, *Dunaliella tertiolecta*, cheese whey effluents, exopolysaccharides, circular economy

## Abstract

The rapid growth of the dairy industry has resulted in a significant increase in the generation of effluents, which are characterized by a high organic content that poses environmental challenges. In alignment with sustainable practices and the principles of the circular economy, this study investigates the valorization of cheese whey (CW) effluents through the cultivation of the microalga *Dunaliella tertiolecta* under mixotrophic conditions. The research aims to utilize cheese whey effluents as a supplemental growth medium to enhance the production of algal biomass and extracellular polymeric substances (EPSs). The results reveal that CW facilitated a 37% improvement in *D. tertiolecta* growth and led to an approximately eight times greater biomass productivity compared to under photoautotrophic conditions, while the EPS production increased by 30%. Chemical and techno-functional analyses of the microalgal biomass and EPSs suggest promising applications as natural product additives for the food industry. Biomass derived from photoautotrophic culture demonstrated greater antioxidant activity and total polyphenols content. Additionally, the lipid profile revealed 16 distinct fatty acids. On the other hand, biomass from the mixotrophic culture exhibited higher protein levels and eight fatty acids, indicating the influence of the cultivation mode on the biochemical composition. Regarding the EPSs, mixotrophic cultivation resulted in elevated antioxidant activity and total polyphenols content, as well as higher protein and sugar levels. Furthermore, the EPSs produced under mixotrophic conditions exhibited superior techno-functional properties compared to those of the photoautotrophic culture, making them ideal candidates for use as alternative natural food additives.

## 1. Introduction

The rapid growth in the global human population in recent decades has led to a substantial increase in the demand for food supplies. The dairy industry, one of the most crucial sectors in the food industry, is expected to face growing pressure to expand its production capacity. However, the industry is also a significant source of waste generation, contributing large volumes of byproducts and wastewater that pose environmental challenges [1,2].

By 2031, worldwide milk production is projected to reach 1060 metric tons, with approximately 30% of this milk being processed into dairy products, like cheese. This production generates between 0.2 and 10 L of cheese whey (CW) for every liter of milk processed [3,4]. Approximately 11 million tons of whey are produced worldwide annually, from which 4.5 million tons originate from EU; and the amounts are expected to increase further over the coming years [5]. As a result, the dairy industry, particularly cheese production, ranks among the highest in water demand and effluent generation. Cheese whey effluents from cheese production pose significant environmental and public health risks when improperly disposed of, either into sewage systems or natural water bodies, such as eutrophication [6]. The CW generated from cheese production contains high concentrations of organic compounds, including lactose (42–60 g L^−1^), proteins (1.4–8 g L^−1^), and lipids (0.99–9.44 g L^−1^). Additionally, it contains nitrogen (7–10 g L^−1^) and phosphorus (0–0.43 g L^−1^), which are present in both organic and inorganic forms [1,2,4].

To address the environmental and eventual public health challenges posed by CW, its valorization for the overall sustainability of the sector has become essential and wastewater treatment strategies that align with the principles of circular economy are increasingly in demand. The circular economy framework focuses on resource recovery, minimizing waste, and the efficient use of side streams as raw materials. This approach not only addresses waste management but also promotes the creation of value from industrial byproducts [7]. Given the heterogeneous composition of CW no universal or standardized method exists for its utilization using microorganisms like microalgae [7,8].

Microalgae are primarily utilized in the following two main areas: production of biomass as a biologically active food additive and isolation of active components from biomass for further use [9,10]. Microalgae have emerged as a promising solution for wastewater treatment, a concept first proposed by Ludwig et al. (1951) [11], and since then, various species have been explored for this purpose. Among them, *Dunaliella* species have demonstrated significant efficacy in nutrient assimilation, mitigating eutrophication in aquatic ecosystems and providing an important ecological benefit [12]. This green marine microalga, known for its adaptability to high-salinity environments, holds substantial promise for applications in the food and pharmaceutical industries due to its rich composition of bioactive compounds, including carotenoids, proteins, fatty acids, and β-carotene [13]. While most microalgae are typically photoautotrophic, some species exhibit the ability to grow mixotrophically or heterotrophically in various substrates containing sugars. This growth potential is facilitated by their ability to utilize external carbon sources, such as the lactose present in cheese wastewater, effectively replacing the need for carbon from photosynthetic process. The photoautotrophic microalga *Dunaliella tertiolecta* meets the requirements for mixotrophic growth in lactose-containing substrates, such as CW. This is due to its ability to produce the enzyme β-galactosidase, which enables it to metabolize lactose as a carbon source that supports its growth under mixotrophic conditions [7,14].

In addition to their role in wastewater treatment, microalgae’s rich nutrient profile makes them a valuable and sustainable food source. Microalgae are abundant in proteins, lipids, polyunsaturated fatty acids, vitamins, pigments, and polysaccharides, exhibiting a variety of bioactive properties, including antibacterial, antioxidant, anticancer, and antiviral properties. These bioactive compounds make microalgae biomass suitable for a wide range of applications, especially in the production of functional foods [9,10].

Among the bioactive compounds produced by microalgae, extracellular polymeric substances (EPSs) hold significant potential for use in the food industry, offering a natural alternative to synthetic additives. EPSs are macromolecules with diverse chemical compositions and structures, which vary depending on the microalgal species from which they originate. The primary components that synthesize EPSs are polysaccharides accounting for 45–95% and proteins with a range from 0.5% to 16.9%, with smaller amounts of lipids, nucleic acid and other molecules [15,16,17]. Microalgae have the ability to secrete these bioactive polymers into the cultivation substrate, forming a protective mucilaginous layer around the cells, especially in response to environmental stress [15,18]. In addition to the soluble EPSs (S-EPSs) found in the culture medium, EPSs can also bind to the microalgal cells. These bound EPSs are categorized into the following two types: loosely bound EPSs (LB-EPSs) and tightly bound EPSs (TB-EPSs) depending on the strength of their attachment to the cells [16]. EPS production is a natural process in microalgae, but certain factors can stimulate enhanced production. For instance, transitioning from photoautotrophic to mixotrophic cultivation, growing microalgae in wastewater, and increasing carbon levels in the culture media can all trigger increased EPS synthesis. The functional properties of EPSs make them ideal for application in the food industry, as a substitute for synthetic additives. EPSs exhibit desirable rheological and emulsifying capabilities, as well as water- and oil-binding properties. Utilizing EPSs in food products aligns with the growing consumer demand for “clean-labeled” products, which focus on natural ingredients [16,17,19,20].

Current research on the cultivation of *D. tertiolecta* under mixotrophic conditions has primarily focused on enhancing growth [21,22]. However, to date, no studies have specifically investigated the impact of cultivation with CW on the production of EPSs.

This current study aimed to improve biomass production and the formation of extracellular polymeric molecules by investigating the use of cheese whey effluents as a supplemental culture medium for the microalga *D. tertiolecta*. The main objective was to further investigate the chemical and techno-functional properties of both EPSs and microalgal biomass, with an emphasis on possible uses as a natural and alternative product addition in the food industry.

## 2. Results

### 2.1. Selection of Optimal CW Concentration

**Figure 1:**
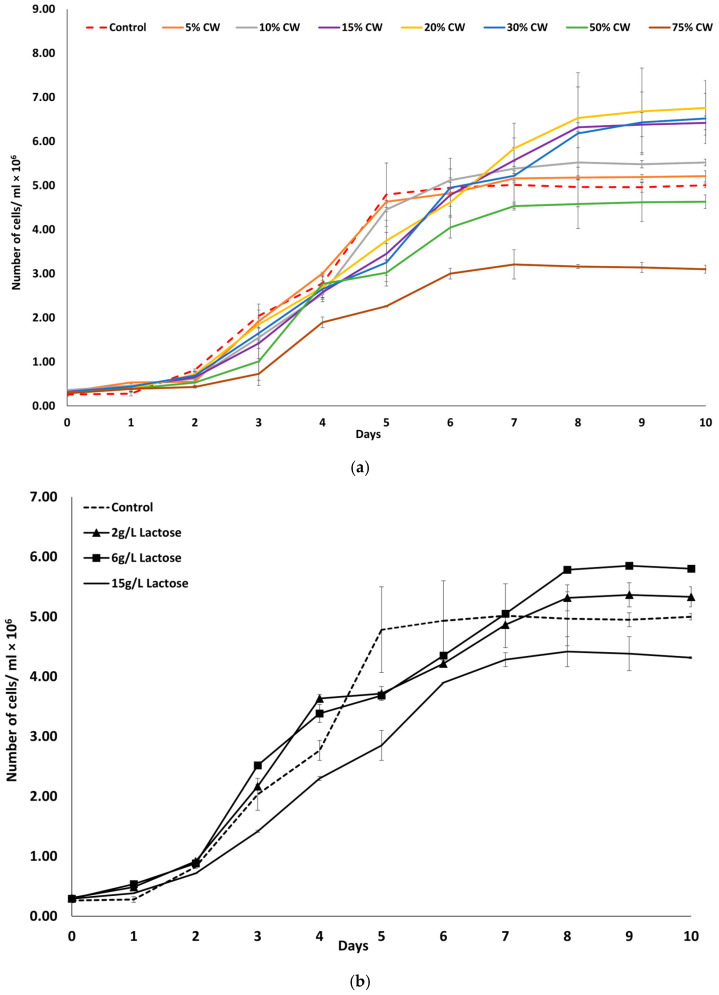
Growth rate as the number of *D. tertiolecta* cells in various concentrations of CW (0–75%) (**a**) and lactose (2 g L^−1^, 6 g L^−1^, 15 g L^−1^) (**b**). The error bars represent the standard deviation of the means, with *n* = 3.

Preliminary experiments were conducted to determine the optimal CW concentration for supporting *D. tertiolecta* growth. Various CW concentrations (0%, 5%, 10%, 15%, 20%, 30%, 50%, and 75%) were tested, and cell growth was monitored over a 10-day period (Figure 1a). The results show that growth was significantly influenced by CW concentration. The highest cell densities were observed at 15%, 20%, and 30% CW, while 75% CW led to notably lower growth. Lower concentrations (5%, 10%, and 50%) supported growth but did not reach the same cell densities as 20% CW. Initially, the growth rate, defined as the number of cells, was similar for both mixotrophic (5–75% CW) and photoautotrophic cultures (0%), referred to as the Control, until day 4. However, from day 5 onward, a notable difference in growth was observed. Specifically, in the photoautotrophic cultures (PCs), the exponential growth phase was inhibited after 5 days, entering a stationary phase. The 20% CW condition appeared to be optimal, providing high cell yields of 6.76 × 10^6^ ± 0.62 cells mL^−1^ compared to the PC which reached 5.00 × 10^6^ ± 0.06 cells mL^−1^ after 10 days. Cultures supplemented with 75% CW exhibited minimal growth, with only a slight increase in cell count before reaching the stationary phase around day 6, resulting in a low final cell density. Similarly, cultures with 50% CW showed minimal growth but reached a higher final cell number. In contrast, cultures grown with 5% and 10% CW demonstrated moderate growth, surpassing the PC but not reaching the levels observed in cultures with 20% CW. The cultures supplemented with 15% and 30% CW exhibited growth comparable but not exceeding that of the 20% CW cultures.

To further examine the role of lactose in *D. tertiolecta* growth, additional experiments were performed using lactose as the sole carbon source at concentrations of 2 g L^−1^, 6 g L^−1^, and 15 g L^−1^, corresponding to the approximate lactose contents in the 5%, 20%, and 50% CW, respectively (Figure 1b). The results demonstrate that *D. tertiolecta* exhibited the highest growth at 6 g L^−1^ lactose, with lower growths at 2 g L^−1^ and 15 g L^−1^. The results mirror trends observed in the CW experiments, where the 20% CW (containing ~6 g L^−1^ lactose) supported optimal growth.

Based on these observations, all subsequent analyses focused exclusively on cultures grown with 20% CW.

### 2.2. Growth Rate of D. tertiolecta Production of Biomass and Extracellular Polymeric Substances

The amount of biomass produced by the MC appeared to be higher compared to the PC. This increase aligns with the growth trends presented in Figure 1. Table 1 highlights the differences in both the biomass and EPS production between the MC and PC and the growth rate of *D. tertiolecta*.

The growth rate of the PC was 0.29 ± 0.014 day^−1^, while the MC presented a growth rate of about 0.31 ± 0.019 day^−1^. The PC produced 0.30 ± 0.001 g L^−1^ of biomass, while the MC with 20% CW yielded eight times more, at 2.51 ± 0.003 g L^−1^. A considerable increase in extracellular polymeric substances production was also observed in the MC with 2.60 ± 0.002 g L^−1^, compared to 1.99 ± 0.002 g L^−1^ produced by the PC.

### 2.3. Composition of the Cheese Whey

The composition of the CW used for the mixotrophic cultivation of *D. tertiolecta* is presented in Table 2.

### 2.4. Determination of the Lactose Content Reduction

**Figure 2:**
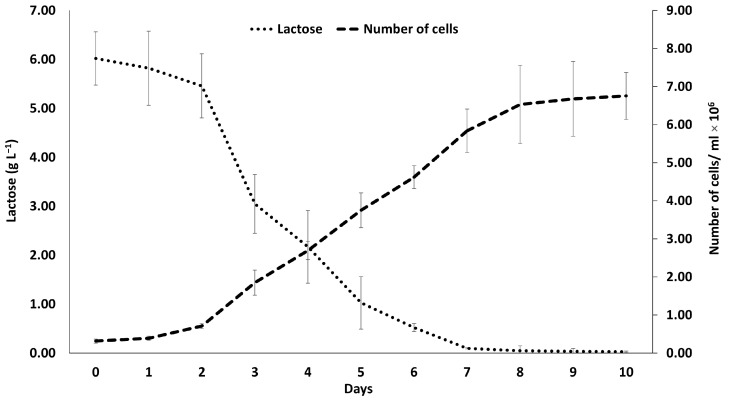
Lactose decreased during *D. tertiolecta* mixotrophic cultivation with 20% CW. The error bars represent the standard deviation of the means, with *n* = 10 (*p* ≤ 0.05).

A gradual decrease in lactose in the MC over the 10-day period was observed (Figure 2). Specifically, the initial lactose concentration was 6.02 g L^−1^. A notable decrease occurred between the 2nd and 3rd days, during which lactose dropped to 3.05 g L^−1^, and by the 7th day of cultivation, the lactose content was completely depleted. Despite this, the microalgal population continued to grow steadily until day 10.

### 2.5. Characterization of the EPSs by FT-IR ATR

**Figure 3:**
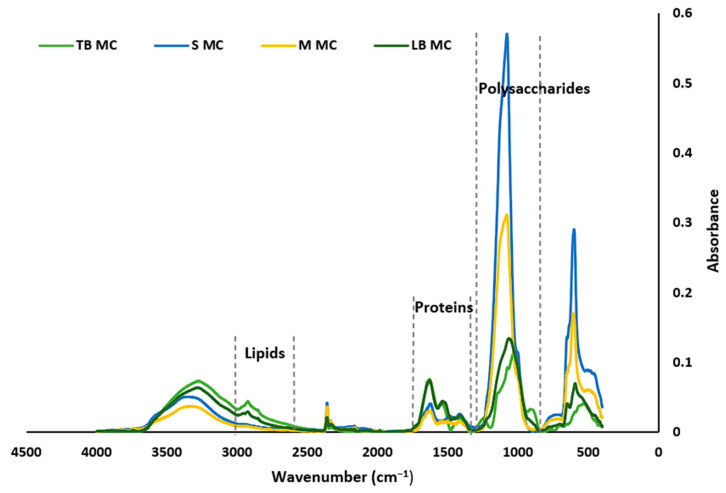
Characterization of the mixotrophic EPS fragments using FT-IR. TB: tightly bound EPSs; S: soluble EPSs; M: mixture of all 3 fragments; LB: loosely bound EPSs.

**Figure 4:**
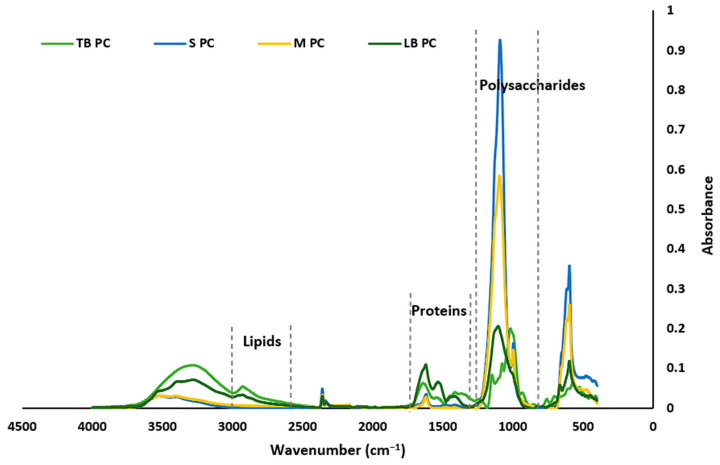
Characterization of photoautotrophic EPS fragments using FT-IR. TB: tightly bound EPS; S: soluble EPS; M: mixture of all 3 fragments; LB: loosely bound EPS.

An FT-IR ATR spectrometer was used to identify the various functional groups contained by the EPSs. It was observed that both types of EPSs (i.e., photoautotrophic and mixotrophic) and their fractions (tightly bound EPS, soluble EPS, a mixture of all three fragments, and loosely bound EPSs) exhibited significant similarities across all the main spectral regions (Figure 3 and Figure 4). The FT-IR spectra in the 900–1300 cm^−1^ region are characteristic of polysaccharide functional groups, while the region between 1500 and 1800 cm^−1^ is indicative of proteins, and the region between 2800 and 3000 cm^−1^ is associated with lipids [23,24]. As observed in both figures, regions characteristic for polysaccharides and proteins are prominent in all four different EPS fragments for both the PC and the MC EPSs. However, regarding lipids, only the LB-EPS and TB-EPS fractions show distinct peaks, whereas the other EPS fractions do not display any significant peaks in this range.

The EPSs derived from both PC and MC exhibited a range of functional groups responsible for their chemical and functional properties. Despite the structural similarities between the EPSs from both cultures, there are notable differences in peak intensities, indicating potential variations in composition and functionality. The peaks at 3317 cm^−1^, 3418 cm^−1^, and 3531 cm^−1^, which are associated with the stretching vibrations of the OH groups, suggest water solubility and water absorbency characteristics of the EPSs [25,26]. The peaks at 2866 cm^−1^ are characteristic of the stretching vibration of the C–H groups of aliphatic hydrocarbons, suggestive of lipid group [24]. The low-intensity sharp peaks at 2363 cm^−1^, 2353 cm^−1^, 2352 cm^−1^, and 2177 cm^−1^ are characteristic of the –C=C and triple –C≡C stretching vibration of the alkyne groups [27,28]. The peak at 1643 cm^−1^ is characteristic of the stretching vibrations of the C=O and C–N (amide I) groups in proteins [23,29,30]. The peak at 1394 cm^−1^ is characteristic of the C–H bending vibration of methyl groups and the stretching vibration of C–N bonds (amide III) [30]. The peak at 1610 cm^−1^ is characteristic of the stretching vibrations of the –C=O and –CHO bonds of the –COOH group in uronic acid [25,31]. The peaks at 1099 cm^−1^, 1076 cm^−1^, and 993 cm^−1^ are characteristic of the stretching vibration of the glycosidic C–O–C bond of polysaccharides [32,33]. The peaks between 599 cm^−1^ and 510 cm^−1^ are characteristic of the bending vibration of the C–X bonds of the alkyl halide group [32,34,35].

The EPSs from both cultivations revealed that polysaccharides consist of alcohols, ketones, aldehydes, ethers, and carboxylic acid functional groups. Proteins, in contrast, are attached to amine, amide, and carboxylic acid functional groups. The prominent absorptions of alcohol and amide groups confirm that polysaccharides and proteins are the major components in the EPSs, influencing their functional properties.

### 2.6. Total Protein Contents of the EPSs and Biomass

**Figure 5:**
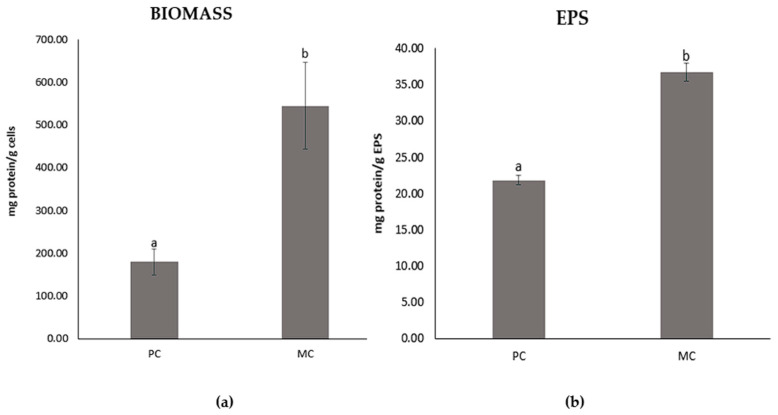
Total protein content of biomass (**a**) and EPSs (**b**). PC: photoautotrophic culture; MC: mixotrophic culture with 20% CW. Values represent the mean ± standard deviation, with *n* = 3. Values bearing different superscripts indicate a statistically significant difference (*p* ≤ 0.05).

The total protein content was remarkably higher in cultures grown with 20% CW, both for the biomass and extracellular polymeric substances (Figure 5). Specifically, the MC’s EPSs (36.69 ± 1.27 mg g^−1^) exhibited 68% greater protein content compared to the PC’s EPSs (21.83 ± 0.71 mg g^−1^). Similarly, the biomass showed a three-fold increase in protein levels, with the PC containing 179.44 ±30.55 mg g^−1^, while the MC achieved 544.46 ± 101.38 mg g^−1^ proteins.

### 2.7. Total Sugar Content of EPS

**Figure 6:**
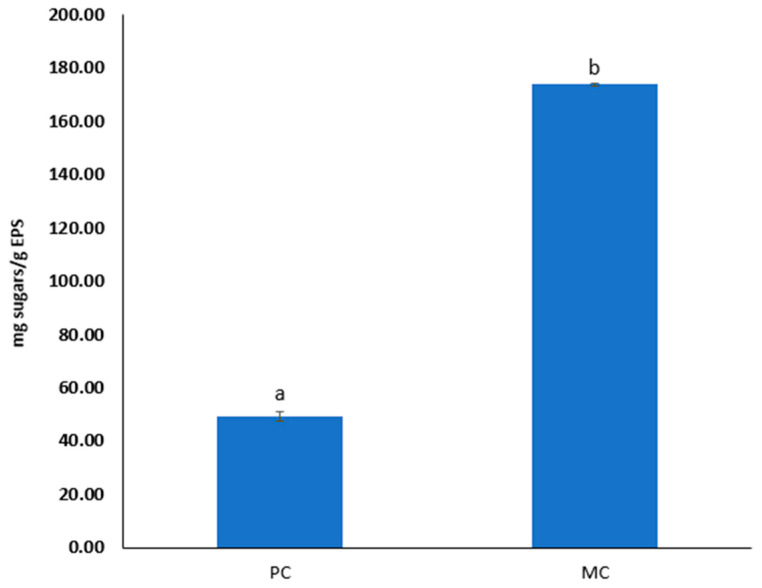
Total sugars contents of the EPSs. PC: photoautotrophic culture; MC: mixotrophic culture with 20% CW. Values represent the mean ± standard deviation, with *n* = 3. Values bearing different superscripts indicate a statistically significant difference (*p* ≤ 0.05).

The difference in total sugar contents of the EPSs from the PC and MC with 20% CW is notably significant, with the former containing 49.12 ± 1.79 mg sugars g^−1^ EPSs and the latter 173.71 ± 0.38 mg sugars g^−1^ EPSs (Figure 6).

### 2.8. Determination of Fatty Acids Profile of the Biomass

The fatty acids profiles of the biomass of *D. tertiolecta* in photoautotrophic cultivation and in mixotrophic cultivation with 20% CW present notable differences between the two cultivation methods (Table 3). The major fatty acids in both conditions were butyric acid (C4:0) and palmitic acid (C16:0), each representing over 10% of the total fatty acids. However, the MC significantly enhanced the proportion of C4:0 (35.57%) compared to the PC (16.39%). The C16:0 content increased slightly in the MC (28.55%) compared to the photoautotrophic growth (27.93%), with no significant difference. Further differences were observed in the composition of omega-3 and omega-6 fatty acids. Linolenic acid (C18:3n3) was considerably higher with the MC (16.56%) than the PC (6.04%). Similarly, linoleic acid (C18:2n6c) increased in the MC (5.32%) compared to the PC (1.8%).

Regarding the overall fatty acids composition, saturated fatty acids (SFAs) were more abundant under photoautotrophic conditions, comprising 68.76% of the total fatty acids, while unsaturated fatty acids (UFAs) constituted 31.24%. Within UFAs, monounsaturated fatty acids (MUFAs) were more prevalent (20.61%) compared to polyunsaturated fatty acids (PUFAs), which made up 10.63%.

Conversely, in the MC, SFAs increased slightly to 70.92%, while the UFAs were lower at 29.07%. Notably, MUFAs reduced significantly under mixotrophic conditions, comprising only 7.19%, whereas PUFAs exhibited a substantial increase to 21.88%.

### 2.9. Antioxidant Capacity of the EPSs and Biomass

**Figure 7:**
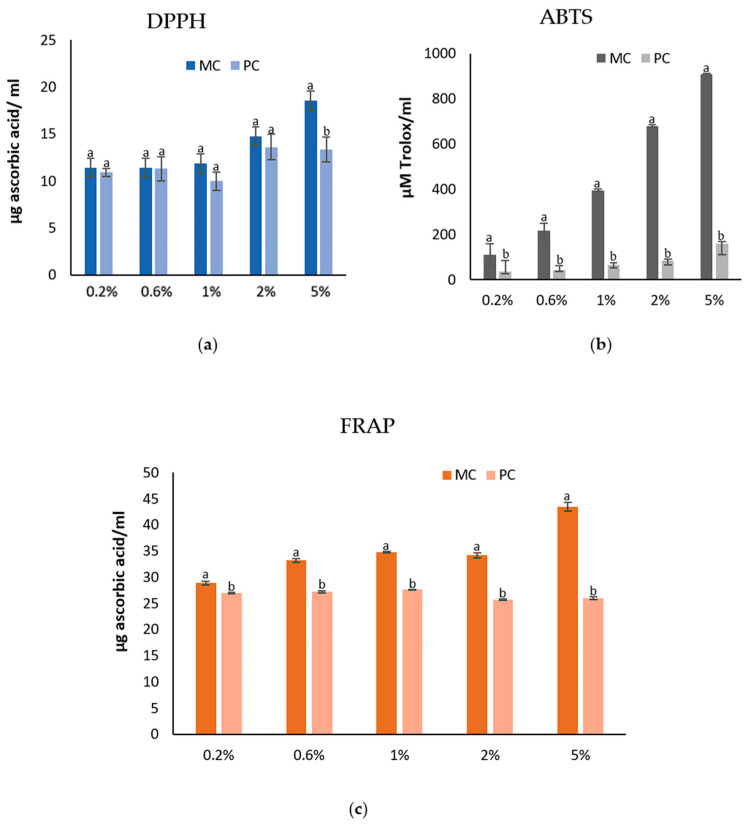
Antioxidant capacities of the EPSs measured with DPPH (**a**), ABTS (**b**), and FRAP (**c**) assays. PC: photoautotrophic culture; MC: mixotrophic culture. Values represent the mean ± standard deviation, with *n* = 3. Values bearing different superscripts indicate a statistically significant difference (*p* ≤ 0.05).

**Figure 8:**
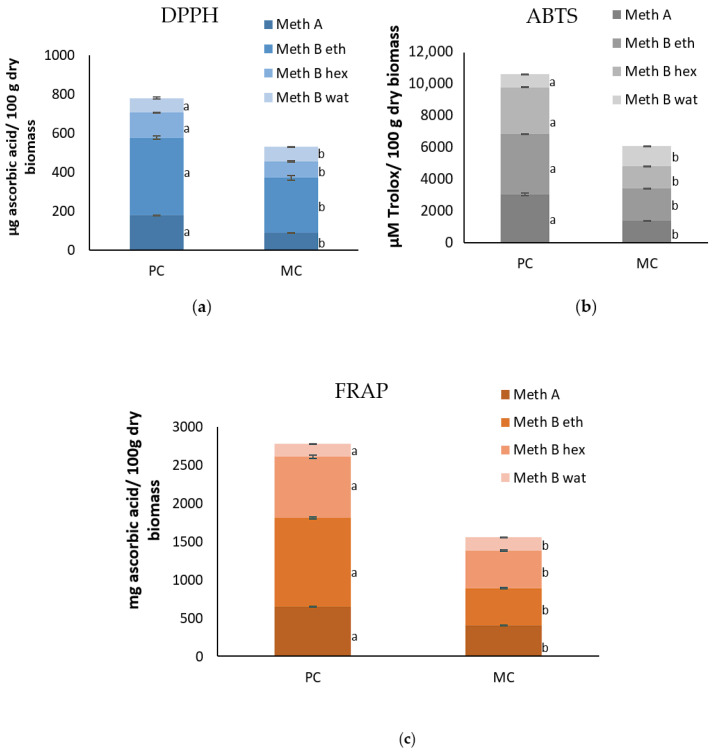
Antioxidant capacities of the biomass measured with DPPH (**a**), ABTS (**b**), and FRAP (**c**) assays. PC: photoautotrophic culture; MC: mixotrophic culture. Values represent the mean ± standard deviation, with *n* = 3. Values bearing different superscripts indicate a statistically significant difference (*p* ≤ 0.05).

The antioxidant capacities of the EPSs and biomass, determined using DPPH, ABTS^+^, and FRAP assays, are presented in Figure 7 and Figure 8. The results indicate that EPSs from the MC consistently exhibited a higher antioxidant capacity compared to the EPSs from the PC, across all different concentrations of the EPSs tested. Conversely, the antioxidant capacity of the biomass demonstrated a different pattern than that of the EPSs. The biomass from the PC consistently exhibited higher antioxidant activity across all four different extracts compared to the biomass from the MC.

Specifically, for the DPPH assay, at the lowest concentration of EPSs (0.2%), the MC showed a value of 11.44 ± 0.58 μg ascorbic acid mL^−1^ while the PC showed 10.92 ± 0.43 μg ascorbic acid mL^−1^. At the 5% EPS concentration, the antioxidant capacity of the MC reached 18.57 ± 1.25 μg ascorbic acid mL^−1^, while for the same concentration, the PC’s value remained lower at 13.32 ± 1.33 μg ascorbic acid mL^−1^. The biomass extracts obtained by method A from the PC yielded 179.38 ± 0.87 mg ascorbic acid/100 g dry biomass, while the MC yielded 88.35 ± 2.08 mg ascorbic acid/100 g dry biomass. A similar pattern was observed for method B, where the photoautotrophic extracts using ethyl acetate and hexane showed 398.13 ± 9.06 and 127.79 ± 2.49 ascorbic acid/100 g dry biomass, respectively, compared to 283.12 ± 12.19 και 84.11 ± 3.28 ascorbic acid/100 g dry biomass for the MC. When deionized water was used as the extraction solvent (method B Wat), the difference in the antioxidant capacities between the PC (75.26 ± 4.09 mg ascorbic acid/100 g dry biomass) and MC (75.22 ± 1.89 mg ascorbic acid/100 g dry biomass) was not as distinct.

A similar trend was observed in the ABTS assay. Specifically, the antioxidant capacity at the lowest concentration of EPSs (0.2%) was significantly higher for the MC (109.5 ± 1.06 μM Trolox mL^−1^) compared to the PC (34.31 ± 2.29 μM Trolox mL^−1^). As the concentration of EPSs increased, this difference became even more definite. For instance, at a 5% EPS concentration, the MC’s EPSs yielded 912.34 ± 4.02 μM Trolox mL^−1^, while the PC yielded 158.81 ± 4.23 μM Trolox mL^−1^. On the other hand, the biomass extracts from the PC consistently exhibited higher antioxidant activities than those from the MC across most extraction methods. For instance, with method A, the extracts from the PC showed a significantly higher antioxidant capacity of 3035.32 ± 93.81 μΜ Trolox/100 g dry biomass compared to 1360.92 ± 6.56 μΜ Trolox/100 g dry biomass from the MC. Similarly, for extracts using ethyl acetate and hexane solvents, the photoautotrophic biomass yielded 3797.98 ± 18.72 and 2950.29 ± 26.27 ascorbic acid/100 g dry biomass, respectively, while the mixotrophic biomass yielded lower values of 2063.76 ± 5.73 and 1398.36 ± 5.73. ascorbic acid/100 g dry biomass. However, the extracts using deionized water as a solvent exhibited the opposite trend, with the MC demonstrating the higher antioxidant capacity (1279.02 ± 6.68) compared to the PC (832.58 ± 19.2).

Subsequently, the antioxidant capacity results for the EPSs, according to the FRAP assay, from the MC, at the lowest concentration (0.2%), demonstrated a value of 28.88 ± 0.36 μg ascorbic acid mL^−1^, while at the same concentration, it was 26.94 ± 0.12 μg ascorbic acid mL^−1^ for the PC. At the highest concentration (5%), the MC’s EPSs showed 43.46 ± 0.82 μg ascorbic acid mL^−1^ compared to 26.00 ± 0.33 μg ascorbic acid mL^−1^ for the PC’s EPS. A noteworthy observation is that the photoautotrophic EPSs reached their peak antioxidant capacity at the 1% concentration (27.60 ± 0.08), after which the values declined slightly as the concentration increased. This suggests that photoautotrophic EPSs may have a concentration-dependent threshold for the optimal antioxidant activity. The biomass from the PC exhibited a higher antioxidant capacity (648.72 ± 5.37 mg ascorbic acid/100 g dry biomass) compared to the biomass from the MC (403.91 ± 3.52 mg ascorbic acid/100 g dry biomass) (method A). Similarly, the extracts from the PC demonstrated much higher antioxidant capacities (method B). The ethyl acetate extract yielded 1157.51 ± 9.87 for the PC, whereas the MC only yielded 487.86 ± 6.33. For the hexane extraction, the PC exhibited 799.71 ± 20.69 while the mixotrophic exhibited 488.73 ± 9.18. However, the extracts using deionized water as a solvent showed the opposite trend, with the MC exhibiting a higher antioxidant capacity (178.28 ± 1.80) compared to the PC (166.12 ± 0.58).

### 2.10. Total Polyphenol Contents of the EPSs and Biomass

**Figure 9:**
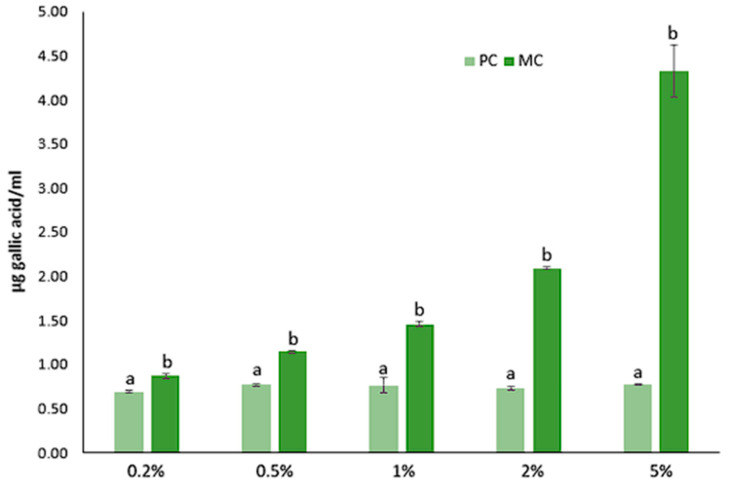
Total polyphenol contents of the EPS solutions. PC: photoautotrophic culture; MC: mixotrophic culture. Values represent the mean ± standard deviation, with *n* = 3. Values bearing different superscripts indicate a statistically significant difference (*p* ≤ 0.05).

The total polyphenol contents (TPCs) of the EPSs derived from both the PC and MC (20% CW) across five different concentrations are presented in Figure 9. In the MC, the TPC of the produced EPSs gradually increased with an increase in the concentration, a trend that was not observed in the photoautotrophic EPSs. Moreover, the mixotrophic EPSs demonstrated a significantly higher polyphenol content than their photoautotrophic counterparts. Specifically, at the highest EPS concentration (5%), the mixotrophic EPSs exhibited 4.32 μg gallic acid mL^−1^, while the photoautotrophic yielded only 0.77 μg gallic acid mL^−1^. At the lowest concentration, the values achieved were 0.87 μg gallic acid mL^−1^ and 0.69 μg gallic acid mL^−1^, respectively.

**Figure 10:**
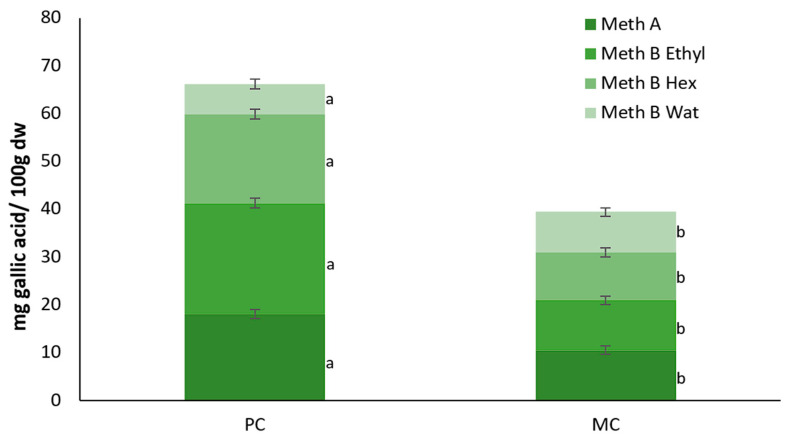
Total polyphenol contents of the biomass. PC: photoautotrophic culture; MC: mixotrophic culture. Values represent the mean ± standard deviation, with *n* = 3. Values bearing different superscripts indicate a statistically significant difference (*p* ≤ 0.05).

On the other hand, the PC biomass generally exhibited a higher TPC, except when water was used as a solvent. Specifically, with method A, the extracts from the PC yielded a higher total polyphenol content of 18.06 ± 0.97 mg gallic acid/100 g dry biomass compared to 10.52 ± 0.89 mg gallic acid/100 g dry biomass from the MC (Figure 10). Similarly, using ethyl acetate and hexane solvents, the photoautotrophic biomass showed higher TPC levels of 23.24 ± 1.86 and 18.58 ± 0.35 mg gallic acid/100 g dry biomass, respectively, while the mixotrophic biomass exhibited lower values of 10.46 ± 0.18 and 11.25 ± 0.12 mg gallic acid/100 g dry biomass. However, when deionized water was used as the solvent, the trend reversed, with the MC showing a higher antioxidant capacity (8.47 ± 0.14 mg gallic acid/100 g dry biomass) compared to the PC (6.32 ± 0.03 mg gallic acid/100 g dry biomass).

### 2.11. Determination of Chlorophyll and Carotenoids Contents of the Biomass

Chlorophyll and carotenoids were found in higher concentrations in the biomass harvested from the PC (Table 4). The difference is remarkable, with the chlorophyll content of the PC at 20.85 ± 0.05 mg g^−1^ compared to 7.31 ± 0.06 mg g^−1^ for the MC. A similar pattern was observed with carotenoids, as the PC exhibited a value of 21.55 ± 0.11 mg g^−1^ compared to 8.07 ± 0.02 mg g^−1^ for the MC.

### 2.12. Physicochemical Properties of the EPS

#### 2.12.1. Foam Capacity and Foam Stability

**Figure 11:**
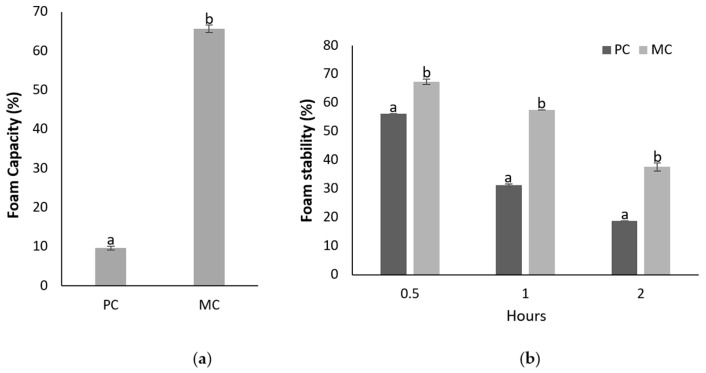
Foam capacity (**a**) and foam stability (**b**) of the EPSs expressed as a %. PC: photoautotrophic culture; MC: mixotrophic culture. Values represent the mean ± standard deviation, with *n* = 3. Values bearing different superscripts indicate a statistically significant difference (*p* ≤ 0.05).

Figure 11 demonstrates the foam capacity and foam stability of the EPSs from the MC and PC, with a clear distinction between the two samples. The foam capacity of the EPSs derived from the MC was significantly higher at 65.68 ± 0.94%, nearly seven times greater than the 9.64 ± 0.47% observed for the EPSs from the PC. This substantial difference indicates that the CW supplementation in the MC significantly enhanced the foaming properties of the EPS.

In addition to the higher foaming capacity, the foam stability of the EPSs from the MC was also superior. After 30 min, the foam stability was 67.25 ± 0.94% for the mixotrophic EPSs, compared to 56.25 ± 0.31% for the photoautotrophic EPSs. This trend persisted even after 2 h, with the foam stability of the mixotrophic EPSs remaining at 37.63 ± 1.41%, while for the photoautotrophic EPSs dropped to 18.75 ± 0%. These results indicate that the EPSs produced from the MC not only generated more foam but also maintained their stability for a longer period compared to the EPSs from the PC.

#### 2.12.2. Emulsification Capacity

**Figure 12:**
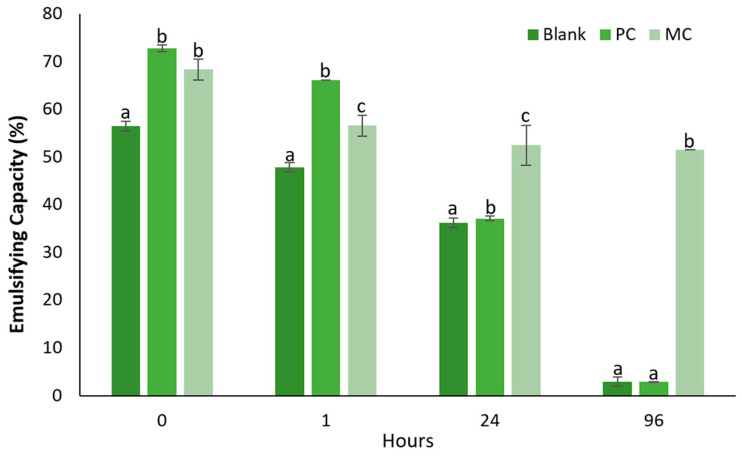
Emulsifying capacity and emulsifying stability of the EPSs expressed as a %. PC: photoautotrophic culture; MC: mixotrophic culture. Values represent the means ± standard deviation, with *n* = 3. Values bearing different superscripts indicate a statistically significant difference (*p* ≤ 0.05).

Emulsifying capacity and emulsifying stability over four timepoints—0 h, 1 h, 24 h, and 96 h—are presented in Figure 12. At timepoint 0 h, the emulsifying capacity of the blank started at 56.52%, while for the photoautotrophic EPSs it was higher at 72.79 ± 1.04% and for the mixotrophic at 68.38 ± 3.12%. After 1 h, the blank’s emulsifying capacity decreased to 47.83%, while the photoautotrophic EPSs showed a smaller drop to 66.18 ± 0%, and the mixotrophic EPSs decreased more significantly to 55.15 ± 3.12%. At this time point, the photoautotrophic EPSs exhibited a higher emulsifying capacity compared to the mixotrophic EPSs. However, after 24 h the emulsifying capacity of both the blank and photoautotrophic EPSs continued to drop, reaching 36.23% and 31.38 ± 0.71%, respectively. In contrast, the mixotrophic EPSs maintained a higher emulsifying capacity at 52.21 ± 3.12%. The most striking difference was observed at 96 h, where both the blank and photoautotrophic EPSs exhibited dramatic reductions in their emulsifying capacity, dropping to 2.90% and 2.92 ± 0.03%, respectively. In contrast, the mixotrophic EPSs remained relatively stable at 51.47%, showcasing their significantly superior emulsifying stability over time. Overall, these results indicate that while the photoautotrophic EPSs initially showed a slightly higher emulsifying capacity at early time points, the mixotrophic EPSs demonstrated a much better long-term emulsifying stability, maintaining its capacity over 96 h.

#### 2.12.3. Determination of Water Holding Capacity (WHC) and Oil Holding Capacity (OHC)

**Figure 13:**
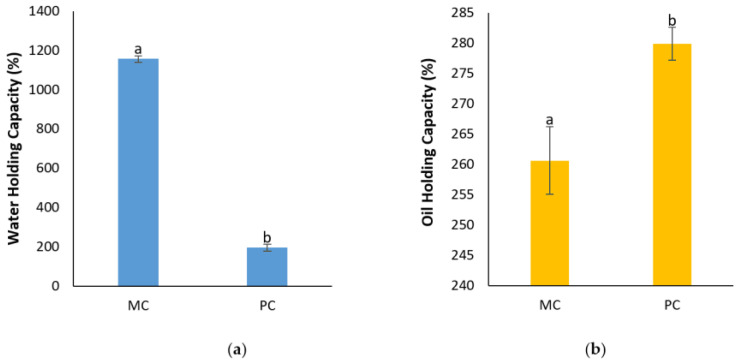
Water holding capacity (**a**) and oil holding capacity (**b**) of the EPSs expressed as a %. PC: photoautotrophic culture; MC: mixotrophic culture. Values represent the mean ± standard deviation, with *n* = 3. Values bearing different superscripts indicate a statistically significant difference (*p* ≤ 0.05).

Figure 13 presents the water holding capacity (WHC) and the oil holding capacity (OHC) of the EPSs from both the MC and PC. The WHC of the mixotrophic EPSs appeared to be significantly higher, reaching 1157.41 ± 15.4% compared to the photoautotrophic EPSs, which only achieved 196.11 ± 17.18%. This demonstrates that the mixotrophic EPSs can hold nearly six times more water than the photoautotrophic EPSs. In contrast, the OHC results indicate that the photoautotrophic EPSs had a slight advantage over the mixotrophic EPSs, with an OHC of 279.91 ± 2.7% compared to 260.63 ± 5.6% for the mixotrophic EPSs, marking about a 7% greater oil-retention capacity for the photoautotrophic EPSs.

#### 2.12.4. Determination of Flocculation Activity

**Figure 14:**
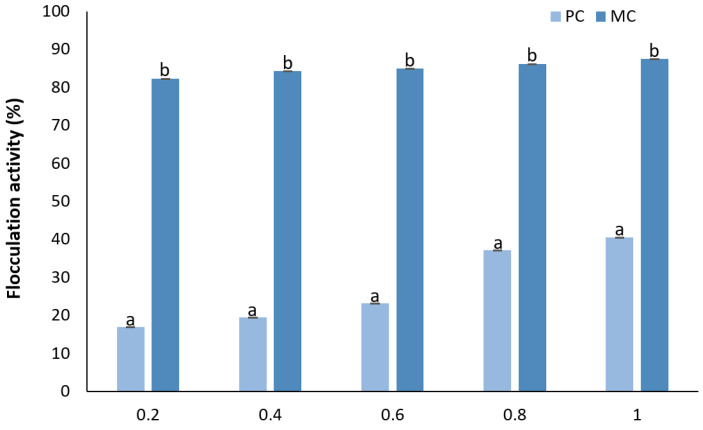
Flocculation capability of the EPSs expressed as a % for the five different concentrations. PC: photoautotrophic culture; MC: mixotrophic culture. Values represent the mean ± standard deviation, with *n* = 3. Values bearing different superscripts indicate a statistically significant difference (*p* ≤ 0.05).

The results of the flocculation activity (FA) show that there was a correlation between the EPS concentration and FA in both cases (Figure 14). At the lowest concentration (0.2 mg mL^−1^), the mixotrophic EPSs demonstrated a significantly higher flocculation ability of 82.32 ± 0.002% compared to 17.02 ± 0.002% for the photoautotrophic EPS. At the highest concentration (1 mg mL^−1^) of EPSs, the mixotrophic EPSs maintained their greater flocculation capability, reaching 87.6 ± 0.005%, while the photoautotrophic EPSs only achieved 40.58 ± 0.002%. This indicates that while both types of EPSs exhibited increases in their flocculation capabilities with an increase in the concentration, with the mixotrophic EPSs consistently exhibiting a much greater flocculation efficiency.

## 3. Discussion

### 3.1. Growth Rate of Culture, Biomass and EPS Production, and Lactose Reduction During Cultivation

The current study aimed to investigate the valorization of CW by utilizing the microalga *D. tertiolecta* for the production of valuable extracellular polysaccharides (EPSs), which hold potential applications in the food industry. Research on enhancing EPS production via the mixotrophic cultivation of *D. tertiolecta* using media supplemented with CW remains limited.

According to the results, *D. tertiolecta* cultures grown on medium containing 20% CW had a 37% higher growth rate than control cultures. This enhanced growth is likely due to the presence of lactose in the CW, which favored microalgal growth, a finding consistent with Zanette et al. (2019) [14]. Their study demonstrated that *D. tertiolecta* cultivated in a substrate containing lactose (5 g L^−1^) produces β-galactosidase, enabling it to metabolize lactose, a valuable source of carbon, which is essential for microalgal growth, thus supporting a higher growth rate. The findings of our study align with this observation, as lactose was significantly reduced during the cultivation period (days 1–6) and was completely depleted by day 7, indicating active consumption by *D. tertiolecta*. Similarly, Andrade et al. (2022) examined *D. tertiolecta* cultivation in dairy industry wastewater containing varying concentrations of lactose, and they found that the MC (2.5 g L^−1^ lactose) exhibited a greater growth rate compared to the PC [21]. However, lactose concentrations exceeding 2.5 g L^−1^ did not support further cell growth, indicating a potential threshold for effective lactose utilization. In contrast, our study demonstrated that *D. tertiolecta* achieved greater yields when cultivated in CW containing 6 g L^−1^ lactose, which did not appear to act as an inhibitory factor. Additionally, Mohammad et al. (2022) examined the growth rate of *D. salina* using CW effluents (20% of substrate) from mozzarella cheese production and reported a higher growth rate under mixotrophic conditions [22]. These findings suggest that *D. tertiolecta* growth can, indeed, be enhanced by CW effluents, but the optimal lactose content may have limitations in promoting growth.

As expected according to the previous results, there was a corresponding increase in the biomass concentration. The biomass derived from the MC increased by eight times compared to the PC. A corresponding performance was observed for the production of extracellular polymeric substances (EPSs), showing that CW favored the production of EPSs with a 30% higher yield. Biomass production was demonstrated to be higher in the MC of *D. tertiolecta,* with the results aligning with Andrade et al. (2022) and Zanette et al. (2019) [14,21]. Casá et al. (2022) also attempted mixotrophic cultivation of *Chlorella vulgaris* using ricotta CW as a substrate, reporting results similar to our findings [36]. This shows that mixotrophic cultivation using CW enhances biomass production. The growth rate of the PC was determined to be μ = 0.29 ± 0.014 day^−1^, while for the MC it was μ = 0.31 ± 0.019 day^−1^, indicating no significant difference between the two. These findings are consistent with the results reported by Velu et al. (2015) [37], who cultivated *D. tertiolecta* using lactose (10 g L^−1^) as a carbon source, observing no significant difference in the maximum growth rates between mixotrophic (μ = 0.23 day^−1^) and photoautotrophic (μ = 0.25 day^−1^) conditions. However, these results contrast with the findings of Zanette et al. (2019), where *D. tertiolecta*, cultivated on a substrate containing lactose (5 g L^−1^), exhibited a significantly higher growth rate in mixotrophic conditions (μ = 0.35 ± 0.04 day^−1^) compared to photoautotrophic conditions (μ = 0.21 ± 0.03 day^−1^) [14,37]. To our knowledge, to date no studies have specifically examined the effect of mixotrophic cultivation of *D. tertiolecta* on EPS production. However, several studies have investigated EPS production under mixotrophic conditions using different microalgal species and carbon sources. A carbon-containing medium promotes the enhanced accumulation of intracellular and soluble extracellular polysaccharides [16]. Trabelsi et al. (2013) explored EPS production from *Arthrospira platensis* cultivated in a glucose-containing substrate and found that MC yielded higher amounts of EPSs compared to under photoautotrophic conditions [18]. Similarly, Zhang et al. (2019) reported that *Chlorella vulgaris*, when grown mixotrophically with glucose, produced higher EPS yields [38]. These studies, along with our findings, suggest that MC is more conducive to increasing EPS production. Although there has not been extensive research on the mechanisms driving increased EPS production in mixotrophic conditions, Xiao and Zheng (2016) proposed that EPSs may function as energy reserves and carbon sinks in response to stress, potentially explaining the higher yields observed under these conditions [15]. Furthermore, Wu et al. (2011) suggested that lactose may play a crucial role as a precursor in the biosynthesis of EPSs, further highlighting its significance in EPS production [39].

### 3.2. Protein and Sugar Contents

As indicated by the results, the presence of CW in the culture medium appeared to enhance both the protein and sugar contents of the biomass and EPS. Notably, the protein content of the biomass from the MC was approximately three times higher than that of the PC. However, the increased protein content observed in biomass cultivated in CW or lactose-containing media is not entirely consistent and may be influenced by several factors. Sánchez-Zurano et al. (2023) and Pereira et al. (2019) conducted studies with different microalgal species, *Chlorella vulgaris* and *Spirulina platensis*, in milk whey and dairy industry wastewater, respectively, and both reported no significant enrichment in protein content [40,41]. In contrast, Abreu et al. (2012) noted higher protein content in mixotrophic cultivation of *C. vulgaris* using dairy effluents [42]. These findings suggest that the protein content may be influenced by both the specific species of microalgae being cultivated and the type of substrate used in the mixotrophic cultivation. Extracellular polymeric substances (EPSs) from MC exhibited an approximately 68% higher protein content compared to those from PC, while the sugar content was enhanced by more than three-fold. The protein content of EPSs from microalgae has not been extensively studied; however, bacterial-derived EPSs have been more thoroughly investigated. Studies show that bacterial EPSs tends to exhibit a higher protein content compared to microalgal EPSs, even when grown under standard conditions or with the addition of whey and glucose [27,43]. Regarding carbohydrate content, the existing literature reports a broad range of sugar content values for bacterial EPSs. For instance, Rehman et al. (2021) documented a lower sugar content in EPSs from three different bacterial isolates than what is observed in our findings [24]. In a study by Solmaz et al. (2018) [43], *Bacillus pseudomycoides* cultivated under standard conditions and with whey supplementation displayed a decrease in EPS sugar content with the addition of whey. Their findings suggest that, under standard conditions, the EPSs exhibited a higher sugar content relative to our results; however, with the addition of whey, the sugar content decreased to comparable levels [43]. Moreover, EPSs from the microalga *Arthrospira platensis* have been reported to contain carbohydrate levels of 23 ± 2.27–39.52 ± 2.34 mg g^−1^ under varying cultivation conditions, significantly lower than the 173.71 ± 0.38 mg g^−1^ found in our study [44]. These observations underscore that both the type of microorganism, whether microalgal or bacterial, and the specific cultivation conditions are critical in determining the biochemical composition of EPSs.

### 3.3. Fatty Acids Profile of the Biomass

Our findings indicate that PUFAs were more abundant in the biomass from the MC, while MUFAs were more prevalent with the PC. Additionally, ω-3 and ω-6 fatty acids were higher with the MC, whereas ω-9 was more prominent with the PC. These results are consistent with previous studies, such as by Lee et al. (2014), who cultivated *D. tertiolecta* under stress conditions and observed variations in the composition of PUFAs, MUFAs, and SFAs [45]. Similarly, Chen et al. (2011) examined the effect of nitrogen starvation on the fatty acids composition of *D. tertiolecta* and found no significant differences, highlighting that environmental conditions, rather than nutritional availability alone, might influence fatty acids profile [46]. Furthermore, our findings are aligned with Lari et al. (2019), who demonstrated that the composition of fatty acids, as well as the percentage of each type, changes according to the trophic mode of cultivation [47]. Similar findings were observed when *Dunaliella salina* was cultivated mixotrophically with fucoidan, an extracted oligosaccharide from brown seaweed, as a carbon source. In that study, saturated fatty acids (SFAs) were more abundant in the mixotrophic culture, which is consistent with our results. Moreover, monounsaturated fatty acids (MUFAs) were more prevalent in the control cultures, aligning with our findings; however, polyunsaturated fatty acids (PUFAs) were found in higher concentrations in the mixotrophic culture, which contrasts with our results [48]. Also, *D. tertiolecta* cultures subjected to nitrogen starvation exhibited comparable amounts of PUFAs and MUFAs between the control and stressed conditions. Notably, both cultures displayed a higher concentration of PUFAs compared to our study, whereas the MUFA levels were lower than those observed for the photoautotrophic control (PC) but higher than those in the mixotrophic culture (MC) [49]. These results suggest that variations in cultivation conditions, including nutrient availability and light exposure, play a significant role in determining the lipid profiles of microalgal biomass, particularly affecting the balance between MUFAs, PUFAs, and ω-3, ω-6, and ω-9 fatty acids.

### 3.4. Antioxidant Activity and Total Polyphenol Contents of the Biomass and EPS

The antioxidant capacity of *D. tertiolecta* biomass (cells) and EPSs was assessed using the following three different methods: DPPH, ABTS, and FRAP. As anticipated, due to the differences between these methods, the values varied; however, the overall trends and patterns in the data remained consistent. Notably, cells from the PC demonstrated superior antioxidant capacity across all methods. In contrast, the antioxidant capacity of the extracellular polymeric substances from the MC appeared to be significantly higher than that from the PC.

This observed reduction in the antioxidant capacity of biomass grown under mixotrophic conditions is consistent with findings reported in the literature. For example, a study on the mixotrophic cultivation of *Spirulina platensis* in cheese wastewater also reported a decrease in the antioxidant capacity of the biomass [41]. Similarly, Azaman et al. (2017) also found diminished antioxidant capacities, as assessed by FRAP, for the biomass of *Chlorella sorokiniana* and *Chlorella zofingiensis* grown under mixotrophic conditions [50]. In contrast, Parra-Riofrío et al. (2020) observed a notable increase in the antioxidant capacity of *Tetraselmis suecica* biomass when the cultivation conditions shifted from photoautotrophic to heterotrophic [51]. It is suggested that the inhibition of oxidative stress under favorable conditions may lead to the formation of free radicals within cells, which subsequently react and reduce the synthesis of antioxidants. This finding supports the conclusion that applying nutritional stress may not be an efficient approach for enhancing the antioxidant content of microalgal biomass [52].

On the other hand, EPSs produced under mixotrophic cultivation exhibited enhanced antioxidant activity compared to EPSs produced under photoautotrophic conditions. This trend has also been observed in other studies. For instance, *T. suecica* cultivated under heterotrophic conditions demonstrated approximately twice the antioxidant activity compared to photoautotrophic conditions [51]. Similar results were reported for *Porphyridium purpureum*, where EPSs produced under heterotrophic conditions exhibited significantly greater antioxidant capacity [53].

Overall, the findings indicate that while mixotrophic cultivation may reduce the antioxidant capacity of microalgal biomass, it enhances the antioxidant activity of EPS.

The total polyphenol contents (TPCs) of both the biomass and EPSs followed a pattern similar to the antioxidant capacity. In the current study, the use of CW did not appear to enhance the polyphenol concentration in *D. tertiolecta* cells. While Pereira et al. (2019) reported higher TPC levels in *Spirulina plantesis* cultivated in cheese wastewater compared to our findings, they did not observe significant changes in the TPCs between mixotrophic and photoautotrophic conditions [41]. Contrary to our findings, other studies have shown increased TPC levels in microalgae grown under different trophic conditions. For instance, *T. suecica* grown heterotrophically and *Spirulina platensis* cultivated mixotrophically, both exhibited higher TPC compared to PC [51,54]. This suggests that the accumulation of polyphenols in microalgae is influenced by both the species and cultivation conditions, and that *D. tertiolecta* may not respond as favorably to the presence of rich organic substrates like CW in term of polyphenol production.

According to our results the TPC of the microalgal EPSs was significantly lower compared to the bacterial EPSs, underscoring the variability in polyphenol synthesis across different microorganisms. Specifically, Adelekan et al. (2020) [55] reported that lactic acid bacteria produced EPSs with a TPC of 1.41–1.58 mg gallic acid mL^−1^. Specifically, *Lactobacillus acidophilus* yielded 1.41 mg gallic acid mL^−1^, while *Lactobacillus fermentum* reached the highest at 1.58 mg gallic acid mL^−1^ [55]. Similarly, Wang et al. (2011) found that EPSs from *Paenibacillus* sp. exhibited 880 μg gallic mL^−1^ [56]. These values are considerably higher than the TPC levels found in microalgal EPSs (4.32 μg gallic acid mL^−1^), further highlighting that the biochemical synthesis of polyphenols and other bioactive compounds can vary greatly depending on the bacterial source. The higher TPC observed in photoautotrophic biomass, according to our results may be attributed to the excess of oxygen that is generated due to the photosynthesis process. During the photoautotrophic growth process, the generation of excess oxygen, combined with nutrients limitation, is known that trigger the synthesis and accumulation of phenolic compounds, which act as a defensive mechanism against oxidative stress [51]. This aligns with the observation that the PC exhibits more robust photosynthetic activity, as evidenced by the higher chlorophyll content compared to the MC.

In the MC, where CW is used as a supplementary medium, there is an abundance of nutrients, which can reduce the oxidative stress typically caused by nutrient deprivation. The availability of these additional nutrients likely decreases the need for the microalgae to synthesize polyphenols as a protective measure.

### 3.5. Chlorophyll and Carotenoids Contents of Biomass

Both photosynthetic pigments, chlorophyll and carotenoids, were observed to be at levels more than 2.5 times higher in the PC compared to the MC. This finding is consistent with the results of Sánchez-Zurano et al. (2023), who reported lower chlorophyll levels in *C. vulgaris* when cultivated in a whey substrate under mixotrophic conditions compared to photoautotrophic growth [40]. However, their study also noted a slight increase in the carotenoid content of the MC when the whey content was low. Similarly, Salah et al. (2023) observed decreases in both the chlorophyll and carotenoid contents of *Desmodesmus* sp. when cultivated in cheese wastewater [57]. Ribeiro et al. (2017) also reported comparable findings cultivating *Chlorella protothecoides* under similar conditions using cheese wastewater [58]. These observations suggest that photosynthetic pigment levels are affected by different growth conditions and external stress factors. The decreased content of chlorophyll in the MC grown in rich organic substrates like CW may be due to the disruption of the photosynthetic organelle formation. Additionally, the shift from photoautotrophic to mixotrophic cultivation may reduce the microalgae’s reliance on light for energy, potentially affecting pigment synthesis [20,42].

### 3.6. Functional Properties of EPS

Extracellular polymeric substances derived from microalgae exhibit valuable functional properties, making them suitable for a range of commercial applications in the food industry [20]. The enhanced functional properties observed in the EPSs from mixotrophic cultivation can be attributed to the specific components present in the EPSs, including polysaccharides, which contain methyl and acetyl groups, as well as proteins, both of which are significantly enhanced in these EPSs [15].

#### 3.6.1. Foaming Capacity and Stability

Foaming ability is a critical surface property that influences the consistency and sensory characteristics of food products. The two main factors that determine foam capacity (FC) and foam stability (FS) are the presence of proteins and polysaccharides, which contribute to the foam’s size and stability [59]. In the current study, the EPSs produced under mixotrophic conditions demonstrated an enhanced FC compared to those under photoautotrophic conditions, with the foam remaining stable even after two hours. Similar findings were reported by Shen et al. (2019), where EPSs produced under mixotrophy exhibited greater FC and FS, although the FC and FS appeared to be lower than that observed for *D. tertiolecta* EPSs [59]. While most studies focus on the FC and FS of bacterial EPS, microalgal EPS consistently demonstrated superior properties in both parameters [32,60,61]. The MC conditions appear to enhance the foaming properties of microalgal EPSs, likely due to the increased sugar and protein contents observed under these conditions. The findings for the FC and FS underscore the importance of placing greater emphasis on the utilization of EPSs derived from microalgae.

#### 3.6.2. Emulsifying Capacity and Stability

An emulsion refers to the mixture of two liquids that are normally immiscible, and the emulsifying properties of EPSs are typically measured by emulsion capacity (EC) and emulsion stability (ES). The protein content linked with EPSs is a critical factor influencing the EC, while the presence of hydrophobic groups also plays a significant role in determining the emulsifying properties [59]. In our study, the EPSs derived from MC exhibited a lower EC (68.38%) compared to EPSs from PC (72.79%). However, the mixotrophic EPS demonstrated remarkable ES over time, maintaining 51.47% stability at 96 h, whereas the photoautotrophic EPSs showed significantly lower stability, with only 2.92% after the same duration. Shen et al. (2019) did not report higher EC when comparing mixotrophic and photoautotrophic derived EPSs, although the EC from *Nostoc flagelliforme* was higher compared to *D. tertiolecta* EPS [59]. Ater 30 min, the emulsifying activity of the EPSs produced by the microalgae *D. salina* was also reported. The emulsifying activity ranged from 66.37 to 85.76% for EPSs produced by *D. salina* cultured under salt stress with 5 M and 0.5 M NaCl, respectively [34]. In comparison, bacterial EPSs, such as those from *Virgibacillus salarius* and *Lactobacillus* sp. have demonstrated high EC and ES. The EC of *Virgibacillus salarius* reached values similar to those of microalgal EPSs, while *Lactobacillus* sp. exhibited significantly higher EC and ES, further emphasizing the diversity in emulsifying properties among bacterial sources [18,32].

#### 3.6.3. Water Holding Capacity & Oil Holding Capacity

Water Holding Capacity (WHC) and Oil Holding Capacity (OHC) are among the most crucial functional properties in food processing, as they play important roles in influencing the texture and consistency of food products. WHC and OHC are key attributes of natural polymeric substances, with WHC largely dependent on factors like particle size, ionic forms, molecular weight, and biopolymer composition. OHC, on the other hand, is significantly influenced by the chemical composition of the biopolymers, through surface porosity [33,59].

In our study, the mixotrophic EPSs exhibited a higher WHC, whereas the photoautotrophic EPSs showed a greater OHC. Both the WHC and OHC values for the EPS from these two cultures appeared to be higher compared to bacterial EPSs from *Macrococcus brunensis* [35]. Jiang et al. (2021) evaluated the WHC of bacterial EPSs from *Enterococcus* sp. and reported that the WHC was lower compared to mixotrophic EPSs, but higher than that of photoautotrophic EPSs, while the OHC was greater for both cultures [33]. Shen et al. (2019) similarly found that mixotrophic EPSs of *N. flagelliforme* exhibited higher WHC and OHC compared to photoautotrophic EPSs [59]. From these observations, it is evident that both the source and cultivation conditions significantly affect the functional properties of EPSs.

#### 3.6.4. Flocculation Activity

Flocculation activity (FA) is a key property of EPSs and is attributed to the presence of carboxyl groups. These groups facilitate binding with divalent cations, such as Ca^2+^, which are then integrated or absorbed by activated carbon, creating complexes with the EPSs [62]. In the current study, the FA of EPSs produced under mixotrophic conditions exhibited higher levels compared to under photoautotrophic conditions. These results are consistent with the findings of Shen et al. (2019), who reported a similar trend, though the mixotrophic EPSs in our study demonstrated greater FA than the corresponding mixotrophic EPSs of *N. flagelliforme* [59]. This enhanced FA also aligns with the findings of Saleem et al. (2021), who showed that bacterial EPSs produced by *Lactobacillus plantarum* followed the same pattern [62]. In their study, they observed that the concentration of EPSs is directly proportional to the FA, with a percentage of 89.5 % for 0.8 mg mL^−1^. The microalgal EPSs from the MC in our experiments showed a comparable FA percentage of 86.32% at the same concentration. Similar results were reported by Kanmani et al. (2011) when evaluating the FA for EPSs derived from the bacterium *Streptococcus phocae*, with an FA percentage 86.4%, closely matching our results for mixotrophic microalgal EPSs [63]. Kanmani et al. (2011) [63] also compared the FA with widely used substances, such as gelatin, xanthan gum, and guar gum. Gelatin exhibited an FA rate of 46.1% at 1 mg mL^−1^, while xanthan gum and guar gum showed higher rates of 76.4% and 58.8%, respectively, at a concentration of 0.6 mg mL^−1^. These values were significantly lower than those of the microalgal EPSs from the mixotrophic culture in our study, which achieved an impressive precipitation rate of 85.06% at the same concentration. From these results, it is evident that microalgal EPSs could serve as an efficient alternative flocculation agent, outperforming many commonly used agents in the food and cosmetic industries.

## 4. Materials and Methods

### 4.1. Microalga Cultures

The marine microalga utilized in this study was *Dunaliella tertiolecta*. The species was obtained from the culture collection of the Laboratory Unit on Harmful Marine Microalgae, School of Biology, Aristotle University of Thessaloniki, which isolated it from marine coastal waters in Greece and performed the identification.

The starting cultures were grown in F/2 medium [64]. The pH and salinity of medium were adjusted to 7.5–7.7 and 3.8%, respectively.

Cultivation was carried out in 1000 mL round flasks under controlled conditions at 21 ± 0.5 °C, using cool white LED lamps (2300 lumens), an air pump with an air flow of 1.5 L min^−1^, and a photoperiod of 18(L):6(D) for 10 days. All nutrient media, pipette tips, and glassware were autoclaved at 121 °C for 15 min prior to use to prevent contamination.

### 4.2. Cheese Whey (CW) and Microalgae Cultivation

Cheese whey effluents was kindly provided by Arvanitis SA, a cheese production company located in Thessaloniki, Greece. The CW originated from the production of feta cheese containing goat and sheep milk. Specifically, the CW was collected from the cheese-making process and stored in polyethylene bottles at −20 °C until use.

#### 4.2.1. Pretreatment of CW

Throughout the experimental process, the CW samples were autoclaved at 121 °C for 15 min to promote protein precipitation. After sterilization, solids were removed by centrifugation at 4000 rpm for 10 min and the supernatant was collected. The upper phase was then filtered using 125 mm filter paper and used for the preparation of the culture medium.

#### 4.2.2. Algae Cultivation with CW

To determine the optimal concentration of CW into F/2 medium for biomass growth and extracellular polymeric substances (EPS) production, different volumes of filtered CW were mixed with F/2 medium (0%, 5%, 10%, 15%, 20%, 30%, 50%, and 75%). Also, three different concentrations of lactose (2 g L^−1^, 6 g L^−1^, and 15 g L^−1^) were examined. These cultures are referred to as the mixotrophic cultures (MCs), while the cultures with 100% F/2 medium were the control cultivations and referred to as photoautotrophic cultures (PCs).

The pH and salinity were adjusted to 7.5–7.7 and 3.8%, respectively, and the growth medium was autoclaved at 121 °C for 15 min to avoid contamination. The medium remained at room temperature for 24 h after autoclaving, before an appropriate amount of microalgae inoculum was added. The cultivation conditions followed were the same as mentioned in Section 4.1.

During a cultivation period of 10 days, pH and cell abundance were monitored daily. The pH was adjusted using a NaOH solution if it dropped below 7.5. The number of cells was monitored using a Neubauer Haemocytometer Chamber and calculated using Equation (1). The results are expressed as the number of cells, mL^−1^ × 10^6^.

(1) $$Total\;cells\;{mL}^{-1}=\frac{Total\;cells\;counted\times Dilution\;factor\times 10,000\;cells/mL}{10^{6}}$$

The microalgae growth rate (*μ*, day^−1^) was calculated using Equation (2), as follows:

(2) $$\mu =\frac{lnN_{2}-lnN_{1}}{t_{2}-t_{1}}$$

where *N*_1_: cell concentrations at the beginning (*t*_1_, day 0), and *N*_2_: cell concentrations at the end (*t*_2_, day 10) of the cultivation period [14].

The mixotrophic cultivation of *D. tertiolecta* with CW was conducted with 10 repetitions, while the photoautotrophic cultivation with 100% F/2 medium was conducted with 3 repetitions.

### 4.3. Biomass Harvest and EPS Extraction

**Figure 15:**
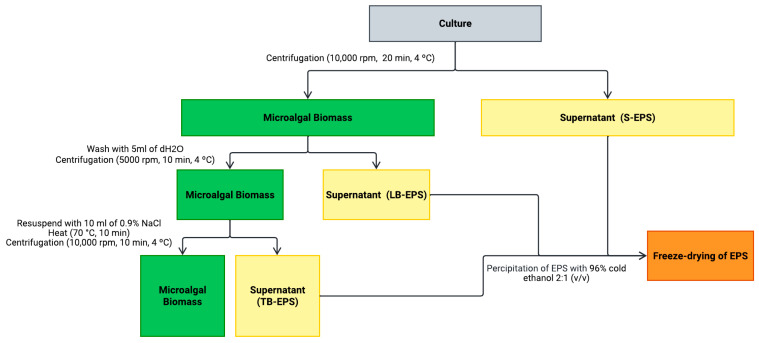
Biomass harvest and EPS extraction process.

Microalgal biomass was harvested according to Ciempiel et al. (2022) [65], with minor modifications (Figure 15). After 10 days of cultivation, the culture medium and cells were separated by centrifugation at 10,000 rpm for 20 min at 4 °C.

Following centrifugation, the following two fractions were collected: the microalgae biomass, which included the loosely bound EPSs (LB-EPSs) and the tightly bound EPSs (TB-EPSs), and the supernatant, which contained the soluble EPSs (S-EPSs).

For the LB-EPSs’ extraction, cells were washed with 5 mL of deionized water and centrifuged at 5000 rpm for 10 min at 4 °C [66]. The remaining cell pellet was resuspended with 10 mL of 0.9% NaCl solution, vortexed, and heated at 70 °C for 10 min. The mixture was then centrifuged at 10,000 rpm for 10 min at 4 °C, with the supernatant containing the TB-EPSs. The remaining cells biomass were freeze dried for further analysis [67].

For the EPS precipitation, cell-free supernatants were mixed with 96% cold ethanol at a 2:1 (*v*/*v*) ratio and left for 24 h at 4 °C according to Ciempiel et al. (2022) [65], with minor modifications. The solutions were then centrifuged at 8000 rpm for 10 min. The resulting EPSs were further washed with 2 mL of 96% cold ethanol and centrifuged again under the same conditions. This washing step was repeated twice using 2 mL of deionized water, followed by centrifugation. The final EPSs obtained were freeze-dried at −55 °C and at a pressure of 0.1 mBar for 48 h, using a Telstar LyoQuest (Telstar, Barcelona, Spain) for further analysis. The S-EPSs, LB-EPSs, and TB-EPSs were freeze-dried separately and maintained in this form solely for the FT-IR analysis. All subsequent analyses and calculations were performed for the combination of the three fragments.

The concentration of dry biomass and EPSs was calculated after the freeze dry process according to Equations (3) and (4).

(3) $$Dry\;Biomass\;(g\;L^{-1})=\frac{Total\;Dry\;Biomass\;(g)}{Total\;culture\;Volume\;(L)}$$

(4) $$EPS\;(g\;L^{-1})=\frac{Total\;Dry\;EPS\;(g)}{Total\;culture\;Volume\;(L)}$$

### 4.4. Determination of the Lactose Content

The concentration of lactose in the culture medium was determined by high-performance liquid chromatography (HPLC). The chromatographic separation was achieved using an Agilent Hi-Plex H (250 mm × 4.6 mm, 8 μm) column in an Dionex UltiMate 3000 HPLC (Thermo Fisher Scientific, Sunnyvale, CA, United States) apparatus equipped with a refractive index detector (RefractoMax 520, ERC, Riemerling, Germany) at 40 °C. A solution of 14 mM H_2_SO_4_ in water was employed for isocratic elution at a flow rate of 0.2 mL min^−1^ [68]. The running time of each sample was 20 min with a 20 µL injection volume and a column temperature at 50 °C. Lactose was identified by retention time and quantified by comparing its peak areas to a linear calibration curve of concentrations (0.01–10 g L^−1^).

### 4.5. Analysis on EPS

#### 4.5.1. Characterization of EPSs with FT-IR

Fourier transform infrared spectrometry (FT-IR) was performed to identify and characterize the functional groups and compounds present in the microalgal EPS. For this analysis all the EPS fragments were used separately.

The FT-IR spectra of the samples were obtained using a Jasco FTIR-6700 (Jasco, Tokyo, Japan) in the frequency range of 4000–400 cm^−1^.

#### 4.5.2. Determination of the Total Protein Contents of the EPSs and Microalgal Biomass

Total protein content was determined using the Bradford method [69]. A standard curve was generated using bovine serum albumin (BSA) (Sigma-Aldrich, St. Louis, MO, USA). To assess the total protein content of the EPSs, a 0.01% aqueous EPS solution was prepared. A total of 1 mL of the EPS solution and 1 mL of the Bradford Assay Solution (TCI, Tokyo, Japan) were mixed and incubated in a water bath for 10 min at 37 °C. The absorbance was then measured at 595 nm.

#### 4.5.3. Determination of the Total Sugar Content of the EPS

The total sugar content of the EPS samples was determined using the phenol-sulfuric acid method [70]. To begin, glucose standard solutions were prepared to create the standard curve. Freeze-dried EPSs were suspended in water to create a 0.1% solution, and a 5% phenol solution was also prepared.

The reaction was initiated by mixing 400 μL of the sample with 400 μL of the 5% phenol solution, followed by the addition of 2 mL of concentrated sulfuric acid. The mixture was vortexed and incubated for 15–20 min at room temperature. The absorbance was measured at 485 nm.

#### 4.5.4. Antioxidant Activity of EPSs

Antioxidant activity was measured in both the EPSs and biomass with DPPH•, FRAP, and ABTS^+^ assays.

The 2,2-Diphenyl-1-picrylhydrazyl (DPPH•) assay was performed according to Gongi et al. (2021), with minor modifications [71]. The standard curve was generated using standard solution of ascorbic acid (10–500 ppm). A control solution containing DPPH• without EPS was also prepared. The sample solutions (EPS) were prepared at concentrations ranging from 0.2–5%. For the assay, 25 μL of each sample solution was mixed with 975 μL of DPPH• solution. The mixture was incubated in the dark, for 30 min at room temperature.

The Ferric-Reducing Antioxidant Power (FRAP) assay was performed according to Deepak et al. (2016), with minor modifications [72]. Initially, the FRAP solution was prepared in a 10:1:1 ratio, consisting of 0.02 Μ FeCl_3_·6H_2_O, 0.01 Μ 2,4,6-Tris(2-pyridyl)-1,3,5-triazine (ΤΡΤΖ) (Sigma-Aldrich, USA) in 0.04 Μ HCl and 0.3 M of acetate buffer (CH_3_ COOH/CH_3_ COONa) at pH 3.6. The standard curve was generated using standard solution of ascorbic acid (10–500 ppm) and a control solution containing FRAP without EPSs was also prepared. Sample solutions (EPSs) were prepared at concentrations ranging from 0.2 to 5%. For the assay, 50 μL of each sample was mixed with 1.45 mL of the FRAP solution and incubated in a water bath at 37 °C for 10 min. Then, the samples were allowed to cool to room temperature before measuring the absorbance at 593 nm. The antioxidant activity is expressed as the μg ascorbic acid per mL of the EPS solution.

2,2′-azinobis-3-ethylbenzothiazoline-6-sulfonic acid (ABTS^+^) assay was performed according to Wang et al. (2021) [31]. The standard curve and samples concentrations were the same as described previously. A control solution containing ABTS^+^ without EPSs was also prepared. For the ABTS solution, 7 mM of 2,2’-azinobis-(3-ethylbenzothiazoline-6-sulfonic acid) (Glentham Life Sciences, Corsham, UK) was mixed with 2.5 mM Κ_2_S_2_O_8_ in a 1:1 ratio and incubated in the dark for 16–18 h at room temperature. For the assay, 50 μL of the sample was added to 950 μL of the ABTS solution and incubated for 30 min in a dark place. The absorbance of the final solution measured at 734 nm, and the antioxidant activity is expressed as the μΜ Trolox per mL of the EPS solution.

#### 4.5.5. Determination of Total Polyphenol Content (TPC)

Total polyphenol content was determined using the Folin and Ciocalteu assay as described by Adelekan et al. (2020), with minor modifications [55]. The standard curve was created using gallic acid (20–200 ppm) as the standard solution, and a control solution without EPSs was also prepared. For the assay, 50 μL of sample solution (0.2–5%) was mixed with 600 μL of deionized water, 50 μL Folin reagent (Sigma Aldrich, USA), and 300 μL of 20% sodium carbonate (NA_2_CO_3_) solution. The samples were incubated at room temperature in a dark place for 1 h, followed by centrifugation at 3000 rpm for 10 min. Absorbance was measured at 725 nm, and the total polyphenol content is expressed as micrograms of gallic acid equivalent (GAE) per mL of the EPS sample.

#### 4.5.6. Physicochemical and Functional Properties of EPS

##### Foaming Capacity and Stability

Foaming capacity and foaming stability were measured according to Zhang et al. (2022) with minor modifications [73]. Initially, 0.5 g of EPS samples were placed in a 50 mL volumetric flask, filled to the mark with deionized water and vortexed until the EPSs were completely dissolved. The solution was then transferred to a cylindrical glass container and stirred using a high-speed disperser (Ultra Turrax T25, IKA Labortechnik, Staufen, Germany) for 3 min at 10,000 rpm. After stirring, the foam volumes (V_0_, V_t_) were measured at 0, 30, and 60 min.

Foaming capacity was calculated using the following formula:

(5) $$FC\;(\%)=\frac{V_{0}-50}{V_{0}}\times 100$$

Foaming stability was calculated using the following formula:

(6) $$FS\;(\%)=\frac{V_{t}-50}{V_{0}-50}\times 100$$

where V_0_: foam volume at time point t = 0 h, and V_t_: foam volume measured after t = 0, 30, 60 min.

##### Determination of Emulsification Capacity

The emulsifying capacity of EPSs was evaluated according to Tarannum et al. (2023), with minor modifications [74]. Briefly, a 2% EPS solution in water (5 mL) was mixed with 5 mL of sunflower oil and vortexed thoroughly for 5 min. A blank sample containing only water and sunflower oil was also prepared. The emulsified layer height (ELH), and the total height (TH) were measured at 0 h to assess the emulsifying capacity and at timepoints of 1, 24, and 96 h to assess the emulsification’s stability.

The emulsification capacity was calculated using the following formula:

(7) $$EC\left(\%\right)=\frac{ELH}{TH}\times 100$$

##### Determination of the Water Holding Capacity (WHC) & Oil Holding Capacity (OHC)

The water holding capacity (WHC) and oil holding capacity (OHC) of the EPSs were evaluated according to Gan et al. (2020), with modifications [75].

Water Holding Capacity (WHC)

Initially, 0.5 g of EPSs was suspended in 20 mL of deionized water. The mixture was allowed to stand at room temperature for 24 h followed by centrifugation at 4000× *g* for 10 min. The liquid phase was discarded, and the weight of the residue was recorded.

WHC was calculated using the following formula:

(8) $$WHC\;(g/g)=\frac{W_{2}-W_{1}}{W_{1}}\times 100$$

where W_1_: initial weight of EPS sample before hydration, and W_2_: final weight of EPS sample after discarding water.

Oil Holding Capacity (OHC)

A total of 0.5 g of EPS sample were mixed with 10 mL of vegetable oil (sunflower oil). The mixture was allowed to stand at room temperature for 1 h followed by centrifugation at 4000× *g* for 10 min. The liquid phase was discarded, and the weight of the residue was recorded.

OHC was calculated using the following formula:

(9) $$OHC\;(g/g)=\frac{O_{2}-O_{1}}{O_{1}}\times 100$$

where O_1_: initial weight of EPS sample before adding vegetable oil, and O_2_: final weight of EPS sample after discarding vegetable oil.

##### Flocculation Activity of EPS

The flocculation activity of the EPSs was evaluated according to Kanmani and Yuvapriya (2018), with minor modifications [76]. Briefly, 0.5 mL portions of the different concentrations of EPSs (0.2–1 mg mL^−1^) were mixed with 0.5 mL CaCl_2_ (6.8 mM) and 10 mL of activated carbon (5 g L^−1^) in a test tube. The blank sample contained all components except EPSs. The mixture was rapidly vortexed for 30 s and allowed to stand at room temperature for 10 min. The absorbance of the supernatant was measured at 550 nm using a spectrophotometer UV-2600 Shimadzu (Shimadzu, Kyoto, Japan).

Flocculation activity was calculated using the following formula:

(10) $$FA\;(\%)=\frac{A-B}{B}\times 100$$

where A: absorbance of samples with EPS, and B: absorbance of blank sample.

### 4.6. Analysis on Microalgal Biomass

#### 4.6.1. Determination of Total Protein Content

For the determination of the total protein content of the cells, the extraction of proteins was necessary. The extraction of proteins was performed according Andreeva et al. (2021), 10 mL of 0.5 M sodium hydroxide solution was mixed with 10 mg of dry microalgal biomass and kept in a water bath for 10 min at 80 °C [77]. The solution was centrifuged at 3900 rpm for 20 min. After that, 1 mL of supernatant mixed with 1 mL of Bradford Assay Solution and kept in a water bath for 10 min at 37 °C. The absorbance was measured at 595 nm. The standard curve that was used is the same as for EPSs.

#### 4.6.2. Fatty Acids Profile of Biomass

Fatty acids of microalgal biomass were extracted and determined according to the direct FAME synthesis method, as described by O’Fallon et al. (2007) [78]. Fatty acid composition was determined using a GCMS-QP2010 Ultra Gas chromatograph mass spectrometer (Shimadzu Europe, Duisburg, Germany) equipped with a TR-CN100 capillary column (100 m × 0.25 mm, 0.20 μm film thickness) (Teknokroma, Barcelona, Spain). The temperature of the injector and the flame ionization detector was set at 250 °C. The oven temperature was initially set at 140 °C for 5 min and slowly increased up to 240 °C with a rate of 4 °C/min and held at this temperature for 30 min. The carrier gas used for the analysis was helium at a flow rate of 20 cm/min. For identification and calibration purposes, the Supelco 37 Component FAME Mix was used (Sigma-Aldrich, St. Louis, MO, United States). The composition of FAMEs was expressed in relative percentage of each fatty acid, calculated by the internal normalization of the chromatographic peak area.

#### 4.6.3. Determination of Chlorophyll and Carotenoids Content

The determination of chlorophyll and carotenoids content requires the extraction of chlorophyll. The extraction was performed according to Oo et al. (2017) with minor modifications using acetone [79]. Briefly, 100 mg of lyophilized biomass were mixed with 80% acetone. Samples remained in sonication bath at 55 °C for 30 min. Afterwards, the liquid centrifuged at 3000 rpm for 10 min. This procedure was repeated until the supernatant had no color. The supernatant was then transferred in a 25 mL volumetric flask and filled up to the mark with 100% methanol to create the final solution. The absorbance was measured using spectrophotometer at different wavelengths of 450, 632, 649, 665, 696, and 750 nm. The final amount of chlorophyll was assessed using the following formulas in Farobie et al. (2023) [80]:

Chlorophyll *a* (μg mL^−1^) = 0.0604 × (A_632_ − A_750_) − 4.5224 × (A_649_ − A_750_) + 13.2969 × (A_665_ − A_750_) − 1.7453 × (A_696_ − A_750_) (11)

Chlorophyll *b* (μg mL^−1^) = −4.1982 × (A_632_ − A_750_) + 25.7205 × (A_649_ − A_750_) − 7.4096 × (A_665_ − A_750_) − 2.7418 × (A_696_ − A_750_) (12)

Chlorophyll *c* (μg mL^−1^) = 28.4593 × (A_632_ − A_750_) − 9.9944 × (A_649_ − A_750_) − 1.9344 × (A_665_ − A_750_) − 1.8093 × (A_696_ − A_750_) (13)

Chlorophyll *d* (μg mL^−1^) = −0.2007 × (A_632_ − A_750_) + 0.0848 × (A_649_ − A_750_) − 0.1909 × (A_665_ − A_750_) + 12.1302 × (A_696_ − A_750_) (14)

Total Chlorophyll (μg mL^−1^) = Chlorophyll *a* + Chlorophyll *b* + Chlorophyll *c* + Chlorophyll *d* (15)

Carotenoid (μg mL^−1^) = A_450_ × 25.2 (16)

#### 4.6.4. Antioxidant Activity and Total Polyphenol Content (TPC) of Microalgal Biomass

For the determination of antioxidant activity and total polyphenol content of the biomass it is necessary to extract the compounds. The extraction of the compounds was achieved as Wang et al. (2023) mentioned using two different methods in order to extract polar and apolar compounds [81].

The first method (Method A) appears to extract polar and apolar compounds in one step using methanol. Briefly, 100 mg lyophilized biomass was mixed with 2 mL of 80% methanol. The samples mixed using vortex and then remained in sonication bath for 30 min. Afterwards, samples were centrifuged at 4400 rpm for 10 min and collected the supernatant. The process was repeated twice, and the final volume of the extract was 6 mL.

The second method (Method B) follows three different steps to extract polar and apolar compounds separately, using hexane (method B Hex), ethyl acetate (method B Ethyl), and hot water (method B Wat). The first step includes mixing 100 mg of lyophilized biomass with 2 mL of hexane. The samples mixed using vortex and then remained in sonication bath for 30 min. Afterwards, samples were centrifuged at 4400 rpm for 10 min and collected the supernatant. The process was repeated two more times, and the final volume of the extract was 6 mL. Following the second step, the residues are mixed with 2 mL of hexane, and the process followed that mentioned above. Finally, the third step of extraction involves the use of hot water (80 °C), which was mixed with the residues following the same process. The extracts from the two different methods were kept at 4 °C until their use.

The determination of the antioxidant activity and total polyphenol content was performed using the two different extracts mentioned in Section 4.5.4 and Section 4.5.5. The extracts were used without further treatment or dilution.

### 4.7. Statistical Analysis

For each assay, measurements were performed in triplicate, and data are expressed as the mean ± standard error (*n* = 3). The results were processed using Microsoft Office, Excel 2021 software. The statistical analysis was conducted using an unpaired *t*-test (GraphPad 9.0, SanDiego, CA, USA), with a significance level of *p* ≤ 0.05.

## 5. Conclusions

In conclusion, the current study demonstrates the significant potential of cheese whey effluents as substrates to enhance the biomass and extracellular polymeric substances (EPS) production of the microalga *Dunaliella tertiolecta* under mixotrophic conditions. The presence of CW favored biochemical compounds production, especially EPS, exhibiting superior biochemical and techno-functional properties. This study offers significant insights into the dual advantages of promoting both microalgal growth and EPS production through the use of cheese whey. These findings present promising opportunities for the development of natural food additives and more sustainable food processing practices, aligning with consumer preferences for clean-label products. Additionally, this approach contributes to closing a vital loop in the dairy industry’s waste management cycle. The results also highlight the necessity of further research into EPSs derived from microalgae, an area that remains relatively underexplored in the current body of scientific literature.

## Figures and Tables

**Table 1:** Growth rate of *D. tertiolecta* biomass and EPS concentration of photoautotrophic (PC) and mixotrophic (MC) cultures.

	Growth Rate (day^−1^)	Dry Biomass (g L^−1^)	EPSs (g L^−1^)
PC	0.29 ^a^ ± 0.014	0.30 ^a^ ± 0.001	1.99 ^a^ ± 0.002
MC (20% CW)	0.31 ^a^ ± 0.019	2.51 ^b^ ± 0.003	2.60 ^b^ ± 0.002

Values represent the mean ± standard deviation, with *n* = 3 for the photoautotrophic culture (PC) and *n* = 10 for the mixotrophic culture (MC). Values in the same column bearing different superscripts indicate a statistically significant difference (*p* ≤ 0.05).

**Table 2:** Composition of the cheese whey.

Material	Result (%)
Proteins	1.37
Sugars	4.20
Fat	0.25
Carbohydrates	4.29
Dry matter	6.54
Moisture	93.46
Crude ash	0.63
Total dietary fibers	<0.30
Lactose	2.96

**Table 3:** Fatty acid composition (%) of *D. tertiolecta* under photoautotrophic (PC) and mixotrophic conditions (MC).

Fatty Acid	PC	MC (20% CW)
C4:0	16.39 ± 0.36	35.57 ± 1.24
C6:0	4.66 ± 0.15	0.00 ± 0.00
C8:0	3.75 ± 0.08	0.00 ± 0.00
C10:0	5.35 ± 0.1	5.51 ± 0.15
C12:0	2.28 ± 0.02	0.00 ± 0.00
C13:0	0.00 ± 0.00	1.29 ± 0.05
C14:0	4.38 ± 0.12	0.00 ± 0.00
C15:1	0.00 ± 0.00	2.37 ± 0.08
C16:0	27.93 ± 0.90	28.55 ± 0.80
C16:1	7.79 ± 0.12	0.00 ± 0.00
C17:1	4.56 ± 0.08	0.00 ± 0.00
C18:0	4.02 ± 0.05	0.00 ± 0.00
C18:1n9c	8.26 ± 0.06	4.82 ± 0.06
C18:2n6t	2.79 ± 0.02	0.00 ± 0.00
C18:2n6c	1.8 ± 0.02	5.32 ± 0.08
C18:3n3	6.04 ± 0.09	16.56 ± 1.08
SFAs ^a^	68.76 ± 1.78	70.92 ± 2.24
UFAs ^b^	31.24 ± 0.39	29.07 ± 1.3
MUFAs ^c^	20.61 ± 0.26	7.19 ± 0.14
PUFAs ^d^	10.63 ± 0.13	21.88 ± 1.16

^a^ Percentage of saturated fatty acids. ^b^ Percentage of unsaturated fatty acids. ^c^ Percentage of monounsaturated fatty acids. ^d^ Percentage of polyunsaturated fatty acids.

**Table 4:** Chlorophyll and carotenoids contents of the biomass of the photoautotrophic (PC) and mixotrophic (MC) cultures.

	Chlorophyll (mg g^−1^)	Carotenoids (mg g^−1^)
PC	20.85 ^a^ ± 0.05	21.55 ^a^ ± 0.11
MC (20% CW)	7.31 ^b^ ± 0.06	8.07 ^b^ ± 0.02

Values represent the mean ± standard deviation, with *n* = 3. PC: photoautotrophic culture; MC: mixotrophic culture. Values in the same column bearing different superscripts indicate a statistically significant difference (*p* ≤ 0.05).

## Data Availability

All data generated or analyzed during this study are included in this article.
